# Unique GNSS data from the Eastern Betic Tectonic Arc (Western Mediterranean), covering almost 28 years of campaign observations

**DOI:** 10.1016/j.dib.2026.112949

**Published:** 2026-06-11

**Authors:** Giorgi Khazaradze, Pedro Alfaro, Francisco Juan García-Tortosa, Antonio Gil-Cruz, Noemi Jacobo-Quiñones, Iván Martin-Rojas, Iván Medina-Cascales, David Rodríguez Collantes

**Affiliations:** a RISKNAT, Geomodels Institute, Department of Earth and Ocean Dynamics, Universitat de Barcelona, Barcelona 08028, Spain; b Departamento de Ciencias de la Tierra y del Medio Ambiente, Universidad de Alicante, c/ San Vicente sn, San Vicente del Raspeig 03690, Spain; c Departamento de Geología, Universidad de Jaén, Campus Las Lagunillas, Jaén 23071, Spain; d Departamento de Ing, Cartográfica, Geodesia y Fotogrametría, Universidad de Jaén, Campus de las Lagunillas, Jaén 23071, Spain; e Centro de Estudios Avanzados en Ciencias de la Tierra, Energía y Medio Ambiente (CEACTEMA), Universidad de Jaén, Campus de las Lagunillas, Jaén 23071, Spain; f Departament de Mineralogia, Petrologia i Geologia Aplicada, Facultat de Ciències de la Terra, Universitat de Barcelona, Barcelona 08028, Spain; g Department of Geophysics, Royal Institute and Observatory of the Spanish Navy (ROA), Plaza de las Tres Marinas S/N, San Fernando, Cádiz 11100, Spain

**Keywords:** GNSS, GPS, RINEX, Eastern Betic Tectonic Arc, Iberian Peninsula

## Abstract

A unique dataset of GNSS observations from the SE Spain covering 28-years of campaign-style observations from 1997 to 2024 is presented. The data are compiled for the geologic study of Eastern Betic Tectonic Arc, where the calculated present-day crustal deformation field shows significant motions ranging from 0 to 2 mm/yr with a dominant NNW-SSE orientation with respect to stable Eurasia. The presented dataset is subdivided into 3 subsets, depending on the authorship. All the data are presented in a standard RINEX: The Receiver Independent Exchange Format, version 2.11. The data interpretation is provided in the accompanying research paper and the data are deposited in Zenodo repository.

Specifications Table

**Table:** 

Subject	Earth & Environmental Sciences
Specific subject area	Space geodesy and present-day crustal deformation studies in the SE Iberian Peninsula, specifically, near the Eastern Betic Tectonic Arc, covering regions of Almeria, Murcia and Alicante in Spain.
Type of data	ASCII files (in standard RINEX 2.11 format), Hatanaka compressed, tarred and gzipped (e.g. UB_GNSS_data_CuaTeNeo-1997-2024.tar.gz).
Data collection	The data were collected during episodic GNSS campaigns starting in 1997 and ending in 2024. First steps of such measurements involve the establishment of the appropriate geodetic monuments. The oldest data in the dataset comes from a CuaTeNeo "*Cuantificación de la Tectónica actual y Neotectónica*" geodetic network, which was established in 1996 by the University of Barcelona (UB) in collaboration with the Institut Cartogràfic i Geològic de Catalunya (ICGC) [3]. It was one of the first regional scale research geodetic networks in Spain, specifically designed to measure active crustal deformation and associated seismic risk. Apart from the CuaTeNeo data, the dataset also includes measurements from the REGENTE network of IGN Spain [10] and GESE-CV (Red Geodésica del SE de la Comunidad Valenciana) network, established by the University of Jaén and University of Alicante. The included data are provided in RINEX (Receiver Independent Exchange) format version 2.11 [4]. They are afterwards compressed using a Hatanaka [5] compression algorithm, which significantly reduced the size of the files. Finally, these Rinex files (e.g. espu2350.97d.gz) are combined using tar software and compressed using gzip tool. Initially, all the files were recorded as raw data, from different GNSS receivers made by Trimble, Topcon and Leica. Each manufacturer uses their native format for the raw data (e.g., TPS for Topcon), making it complicated to use for post-processing. For this reason, it is a standard practice to translate these raw files into a RINEX for public dissemination. The translation from Raw to RINEX format was done using corresponding GNSS receiver manufacturer proprietary software (e.g, DAT2RIN for Trimble) and/or the generic TEQC software [6]. In term of the observation methodology, as a rule, each point was observed during a consecutive 2 or 3-days, at least for 8 h each day. In many cases, the instrument was left recording during the 2 consecutive nights, resulting in 3-day observations of 8-10 h for the 1^st^ and the 3^rd^ day and 24 h for the 2^nd^ day.
Data source location	Data were collected from the SE Spain, from the three geodetic networks specifically designed and built for geodynamic/tectonic studies: CuaTeNeo, REGENTE and GESE-CV (see Fig. 1). They are described in detail in the text of the manuscript where the 3 published datasets are presented. Most of the geodetic monuments were built specifically for detecting slow-deformations of 1 mm/yr and to ensure their long-term stability. Most of them are located on bedrock outcrops, thus not prone to anthropogenic or other non-tectonic deformation processes.
Data accessibility	Repository name: Zenodo Data identification number: https://doi.org/10.5281/zenodo.17666303 Direct URL to data: https://zenodo.org/records/17666304 Instructions for accessing these data: Access to the data is free and governed by Creative Commons Attribution 4.0 International license.
Related research article	Related research article has been submitted to the journal of Geodesy and Geodynamics on 26/12/2025; Manuscript number: GEOG-S-25-00462. On the 16/6/2026 it was re-submiited for publication with minor revisions (GEG-D-25-00285R1). Title: Tectonic salient characterization based on GNSS data: The case of the Eastern Betic Tectonic Arc in the western Mediterranean Authors: Giorgi Khazaradze; Noemi Jacobo-Quiñones; Pedro Alfaro; Iván Medina-Cascales; Francisco Juan García-Tortosa; Antonio J Gil-Cruz; David Rodríguez Collantes, Iván Martin-Rojas

## 1 Value of the Data

• Covers 28 years of observations, which makes their value unique, especially in the region where the deformation rates are slow: below 2 mm/yr.
• The included data has never been published earlier and thus, it is the first time that it is available to the public.
• Constitutes a compilation of the data collected during several dozen campaigns conducted independently by various universities.
• The provided data have been quality checked and to our maximum ability, the metadata provided in the headers of the daily RINEX files, are correct, which includes the correctness of the GNSS receiver and antenna types, as well as the antenna heights, specified in the RINEX headers.
• Other researchers, can re-process these data and improve the deformation velocities (and consequently, their interpretations) published previously.
• Other researchers can plan new observational campaigns in the SE Betics, considering the previous history of the observed points and benefit from the efforts undertaken by us during almost 30 years of filed activities.

## 2 Background

**Fig. 1:**
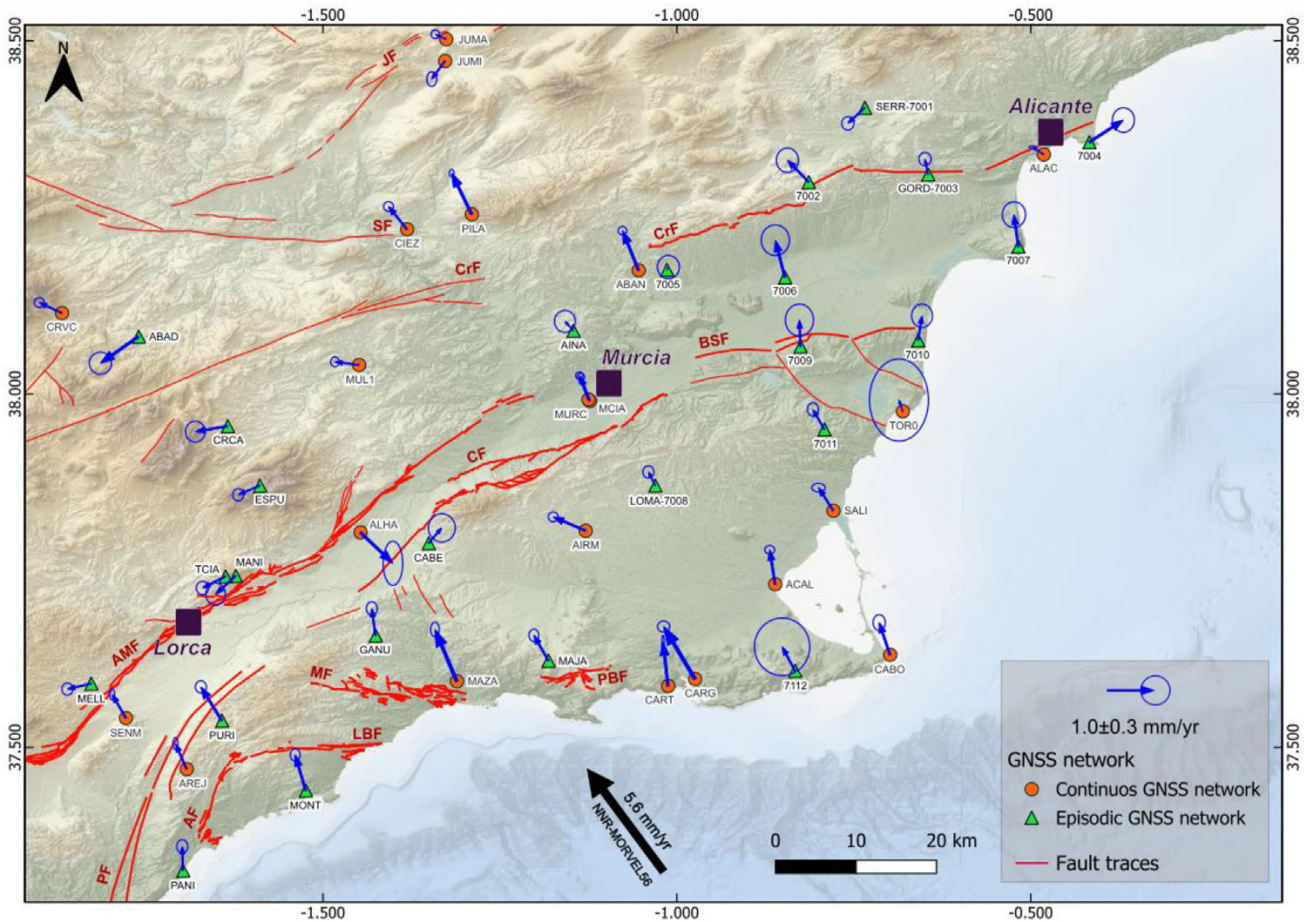
Horizontal crustal deformation velocities from the Eastern Betic Cordillera in SE Spain, derived from the Episodic (green triangles) and Continuous (red circles) GNSS observations from 1997 to 2024. Traces of active faults after [1,9]. North-EBTA faults: CrF= Cevillente Fault and AMF= Alhama de Murcia Fault; Central-EBTA faults: PF= Palomares Fault, CF= Carrascoy Fault, and BSFZ= Bajo Segura Fault Zone; South-EBTA faults: AF= Arejos Fault, LBF= Lomo de Bas Fault, MF= Moreras Fault, and PBF= Peñas Blancas Fault. The figure is adopted from [1].

Several of the most destructive earthquakes that have occurred in Spain over the past two centuries have occurred in the Eastern Betic Cordillera [7], caused by the 5–6 mm/yr NNW–SSE convergence between the Nubia and Eurasia plates (e.g. [8]). In the related research paper [1], the 2D crustal deformation velocities are interpreted. These were derived from the continuous and episodic GNSS data (Fig. 1). This publication only includes the data of the episodic campaigns, since the data from the continuous GNSS stations (e.g., ALAC in Alicante) are available publicly through the operators’ websites (e.g. https://datos-geodesia.ign.es/ERGNSS/).

By publishing these data, we provide a unique opportunity for the interested researchers to take advantage of the work done by us and our predecessors during the past 30 years. This dataset provides a three-decade-long observation history in this relatively slowly deforming yet seismically active and hazardous region of the Iberian Peninsula. Furthermore, the new data presented here constitute a major step forward in understanding the seismic characteristics of this highly populated region and will be used for future seismic hazard assessments.

## 3 Data Description

**Fig. 2:**
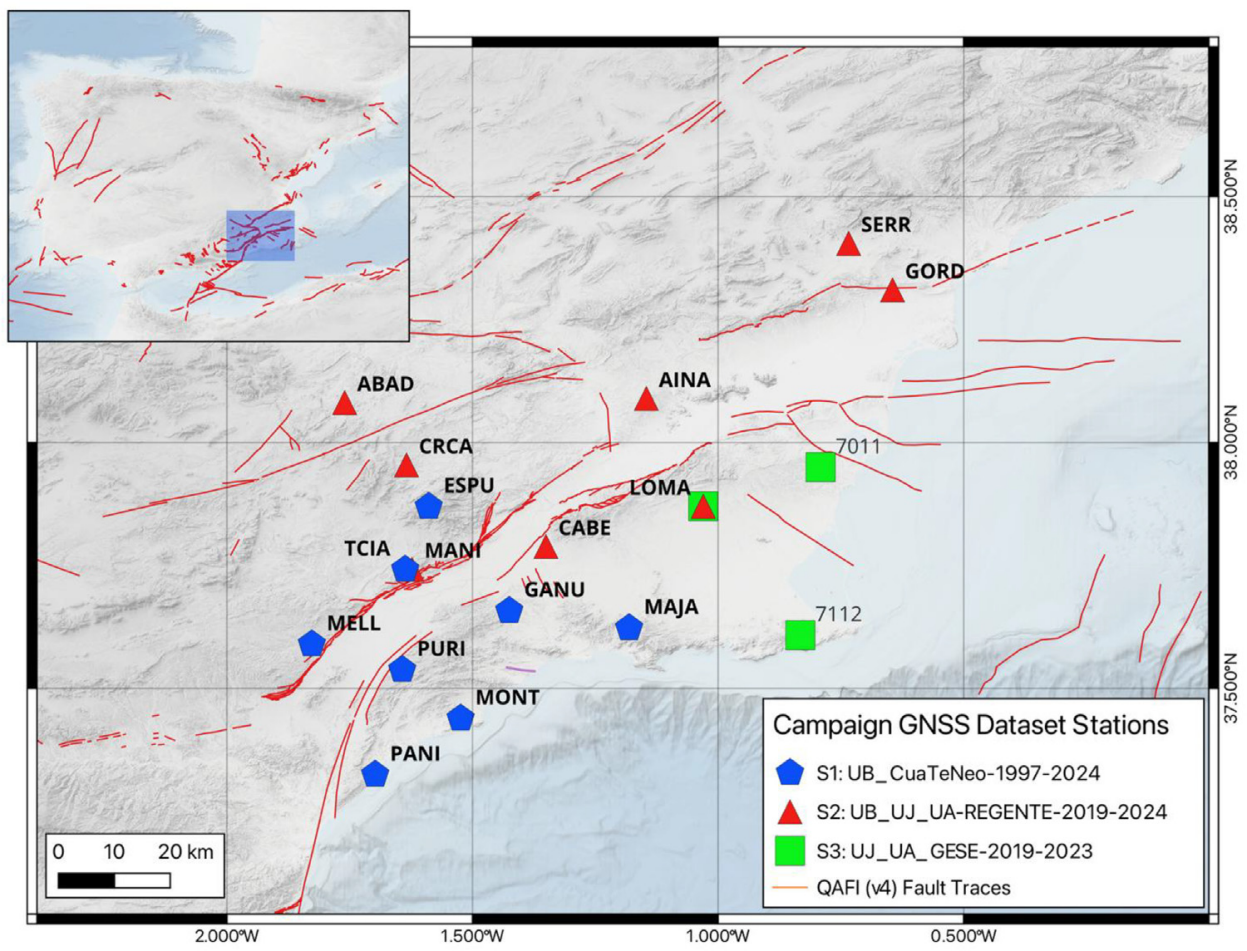
Map with the location of the campaign style GNSS geodetic sites included in the 3 datasets listed in Table 1. The inset shows a location of the study area within the Iberian Peninsula. Red lines show quaternary faults from the QAFI 4.0 database [4,5].

The provided data include the 3 separate datasets (Table 1) with all the campaign style episodic GNSS observations used in the related research paper [1]. The location of the sites is shown in Fig. 1 as triangles and in Fig. 2 using different symbols to differentiate the 3 datasets. All the data are provided in RINEX format V2.11 [4], compressed using Hatanaka [5] compression, and finally then tarred and gzipped. Hence, the extension *.tar.gz.

**Table 1:** List of the provided datasets. # of files shows number of included Rinex files.

#	Dataset	File Name	# files	# stations	File Size
1	S1	UB_CuaTeNeo-8stas-1997-2024.tar.gz	184	8	48.0 Mb
2	S2	UB_UJ_UA_REGENTE-2009-2024.tar.gz	56	8	34.0 Mb
3	S3	UJ_UA_GESE-CV_2019-2023.tar.gz	54	3	52.0 Mb

Al the RINEX files were obtained from the raw observations, from different type GNSS receivers made by Trimble, Topcon and Leica. Each company uses their native format (e.g. TPS for Topcon), which were translated to RINEX using proprietary software of the corresponding manufacturer or TEQC software [6]. Each dataset is described in more detail below.

### 3.1 Dataset S1: UB data from 8 stations of CuaTeNeo network (1997-2024)

Here we provide a dataset with 184 daily RINEX observation files (in Hatanaka format and gzipped) for the 8 stations (Table 2) of the CuaTeNeo geodetic network that were used in the research paper [1]. This network in general consists of 15 geodetic monuments that were established in 1996 by the University of Barcelona (UB) and Catalan Cartographic Institute (ICC) [3], specifically to study tectonic activity in the Eastern Betic Shear Zone in SE Spain. The network was observed for the first time in 1997. Table 2 includes the description of the 8 monuments of the CuaTeNeo network included in this paper.

**Table 2:** Location and the description of the 8 stations of the CuaTeNeo geodetic network monuments used in the related research paper and included in dataset S1.

#	Number	Code	Location	Municipality	Longitude (°)	Latitude (°)	MSLH (m)	Mnmnt. type	Rock Type	Rock Age	Related Faults
1	8001	ESPU	Sierra Espuña	Totana	358.411	37.87	1505	Concrete rect. Base w/ thread	Limestone	Jurassic	Alhama de Murcia
2	8002	TCIA	Sierra de la Tercia	Totana	358.363	37.742	900	Concrete rect. Base w/ thread	Dolomite	Triassic	Alhama de Murcia
3	8003	MELL	Cuesta del Mellado	Lorca	358.173	37.59	575	Concrete rect. Base w/ thread	Sandstones, Quartz	Devonian-Permian	Alhama de Murcia
4	8011	PANI	Cala Panini	Cuevas de Almanzora	358.302	37.325	15	Thread in the rock	Rhyodacite	Miocene	Palomares -Terreros
5	8012	MONT	Montalbán	Águilas	358.476	37.439	45	Concrete rect. Base w/ thread	Phyllite	Cambrian-Triassic	Terreros-Moreras
6	8013	PURI	Casa Reverté	Lorca	358.357	37.538	570	Concrete rect. Base w/ thread	Gneiss	Permian-Triassic	Palomares -Terreros
7	8014	GANU	Sierra de Almenara	Mazarrón	358.575	37.658	390	Concrete rect. Base w/ thread	Serpentine	Permian-Triassic	Terreros-Moreras-Alhama de Murcia
8	8015	MAJA	Colladdo de Majasarte	Mazarrón	358.819	37.623	365	Concrete rect. Base w/ thread	Schist	Paleozoic	Moreras-Alhama de Murcia

The list of individual daily RINEX observation files by year (from 1997 to 2024) is provided in Table 3. Each year corresponds to an individual campaign conducted mainly by the UB group. 1997, 2002 and 2006 in collaboration with *Real Observatorio de la Armada* (ROA) and *Institut Cartogràfic de Catalunya* (ICC), presently called *Institut Cartogràfic i Geològic de Catalunya* (ICGC). Data from the CuaTeNeo stations GANU and MELL for the year 2021 were collected by the *Universidad Politécnica de Madrid* (UPM) and was used in [9].

**Table 3:** List of 184 Rinex files included in the dataset S1 named: “UB_CuaTeNeo-8stas-1997-2024.tar.gz”.

**1997** espu1120.97d.gz ganu1120.97d.gz maja1120.97d.gz mell1120.97d.gz mont1120.97d.gz puri1120.97d.gz tcia1120.97d.gz espu1130.97d.gz ganu1130.97d.gz maja1130.97d.gz mell1130.97d.gz mont1130.97d.gz puri1130.97d.gz tcia1130.97d.gz mell1140.97d.gz mont1140.97d.gz pani1140.97d.gz puri1140.97d.gz tcia1140.97d.gz ganu1150.97d.gz maja1150.97d.gz pani1150.97d.gz espu1160.97d.gz ganu1160.97d.gz maja1160.97d.gz mont1160.97d.gz pani1160.97d.gz tcia1160.97d.gz espu1170.97d.gz mont1170.97d.gz pani1170.97d.gz tcia1170.97d.gz **2002** espu2700.02d.gz ganu2700.02d.gz maja2700.02d.gz pani2700.02d.gz tcia2700.02d.gz espu2710.02d.gz ganu2710.02d.gz maja2710.02d.gz pani2710.02d.gz tcia2710.02d.gz espu2720.02d.gz ganu2720.02d.gz maja2720.02d.gz mell2720.02d.gz mont2720.02d.gz pani2720.02d.gz tcia2720.02d.gz espu2730.02d.gz ganu2730.02d.gz mell2730.02d.gz mont2730.02d.gz espu2740.02d.gz ganu2740.02d.gz mell2740.02d.gz mont2740.02d.gz puri2740.02d.gz ganu2750.02d.gz puri2750.02d.gz ganu2760.02d.gz puri2760.02d.gz **2006** espu2610.06d.gz ganu2610.06d.gz puri2610.06d.gz espu2620.06d.gz ganu2620.06d.gz puri2620.06d.gz espu2630.06d.gz mell2630.06d.gz mont2630.06d.gz puri2630.06d.gz espu2640.06d.gz mell2640.06d.gz mont2640.06d.gz espu2650.06d.gz maja2650.06d.gz mell2650.06d.gz mont2650.06d.gz pani2650.06d.gz puri2650.06d.gz tcia2650.06d.gz espu2660.06d.gz maja2660.06d.gz pani2660.06d.gz puri2660.06d.gz tcia2660.06d.gz espu2670.06d.gz maja2670.06d.gz pani2670.06d.gz tcia2670.06d.gz **2009** mell2600.09d.gz pani2600.09d.gz espu2610.09d.gz mell2610.09d.gz pani2610.09d.gz espu2620.09d.gz mell2620.09d.gz mont2620.09d.gz pani2620.09d.gz tcia2620.09d.gz espu2630.09d.gz mont2630.09d.gz tcia2630.09d.gz espu2640.09d.gz ganu2640.09d.gz mont2640.09d.gz puri2640.09d.gz tcia2640.09d.gz ganu2650.09d.gz puri2650.09d.gz ganu2660.09d.gz **2011** mell1430.11d.gz puri1430.11d.gz tcia1430.11d.gz espu1440.11d.gz ganu1440.11d.gz maja1440.11d.gz mell1440.11d.gz mont1440.11d.gz puri1440.11d.gz tcia1440.11d.gz espu1450.11d.gz ganu1450.11d.gz maja1450.11d.gz mont1450.11d.gz puri1450.11d.gz tcia1450.11d.gz espu1460.11d.gz ganu1460.11d.gz maja1460.11d.gz mell1460.11d.gz mont1460.11d.gz puri1460.11d.gz tcia1460.11d.gz ganu1470.11d.gz maja1470.11d.gz mell1470.11d.gz mont1470.11d.gz **2015** pani3340.15d.gz pani3350.15d.gz **2016** pani3290.16d.gz pani3300.16d.gz pani3310.16d.gz **2017** pani3270.17d.gz pani3290.17d.gz **2018** puri3230.18d.gz puri3240.18d.gz puri3250.18d.gz mont3260.18d.gz pani3260.18d.gz puri3260.18d.gz mont3270.18d.gz pani3270.18d.gz puri3270.18d.gz puri3280.18d.gz **2021** ganu1590.21d.gz ganu1600.21d.gz mell1730.21d.gz mell1740.21d.gz **2022** maja1650.22d.gz maja1660.22d.gz maja1670.22d.gz **2024** maja0550.24d.gz mont0550.24d.gz ganu0560.24d.gz maja0560.24d.gz mont0560.24d.gz pani0560.24d.gz puri0560.24d.gz ganu0570.24d.gz maja0570.24d.gz mont0570.24d.gz pani0570.24d.gz puri0570.24d.gz pani0580.24d.gz espu0650.24d.gz mell0650.24d.gz espu0660.24d.gz mell0660.24d.gz tcia0660.24d.gz espu0670.24d.gz mell0670.24d.gz tcia0670.24d.gz

For the provided 184 RINEX files we performed a quality control (QC) using TEQC software [6]. The results are provided in a file “***UB_CuaTeNeo-8stas_QCstats-1997-2024.txt***”, which contains a typical summary line of the TEQC output for each daily RINEX files listed in Table 3. This includes the basic statistics of the data, including the start and the end time of the observations, their duration and mp1 and mp2 multipath values. Example and the format of the included data is given in Table 4.

**Table 4:** Example of the Quality Check (QC) summary file of the TEQC output.

rnx_fname	STA	DOY	YEAR	MM	DD	MN.SS	YEAR	MM	DD	MN.SS	durat	dt	#expt	#have	%	mp1	mp2	o/slps
espu1120.97o	ESPU	112	1997	4	22	18:21	1997	4	22	23:59	5.633	30	5000	4542	91	0.2	0.4	324
ganu1120.97o	GANU	112	1997	4	22	21:49	1997	4	22	23:59	2.167	30	2017	1824	90	0.2	0.3	1824

Abbreviations: rnx_fname: RINEX file name; STA: Sta 4-char ID; DOY: Day Of the Year; YEAR: Year; MM: Month; DD: Day; MN: *Mins.*; SS: *Secs.*; durat: Duration in *hrs*; dt: Data sampling interval in seconds; #expt: Number of possible observations; #have: Number of complete observations; %: Ratio of complete to possible observations; mp1 and mp2: RMS “multipath combinations” *(*cm*)*; o/slps: Observations per slip.

### 3.2 Dataset S2: UB/UJ-UA data from IGN REGENTE network (2009-2024)

In this section we include a dataset of 56 daily RINEX from the 8 points of the REGENTE network of IGN Spain [10] used in the research paper [1]. Detailed description of these sites is provided in Table 5, Table 6.

**Table 5:** Location and description of the IGN REGENTE network geodetic sites used in the paper. The last column includes a link to the official log-sheets (Reseña Vértice Geodésico) of the sites, which can be obtained from the official IGN search website (https://www.ign.es/web/ign/portal/gds-vertices).

#	Num.	Code	Location	Municipality	Longitude (°)	Latitude (°)	MSLH (m)	Mnmnt. type	Rock Type	Rock Age	Related Faults	Notes
1	91315	**ABAD**	Fuente Abad	Cehegin (Murcia)	358.2398	38.0813	645.5	forced-centering concrete pillar	Conglomerates	Miocene	Crevillente	Regente (91124)
2	91315	**AINA**	Alcaina	Molina de Segura (Murcia)	358.8541	38.0897	339.0	forced-centering concrete pillar	Conglomerates	Miocene	Segura, Carrascoy	Regente (91315)
3	95457	**CABE**	Cabezd.gz Blanco	Alhama de Murcia (Murcia)	358.6497	37.7888	275.6	forced-centering concrete pillar	Conglomerates	Pleistocene	Carrascoy	Regente (95457)
4	93267	**CRCA**	Carcavalar	Mula (Murcia)	358.3657	37.9544	694.8	forced-centering concrete pillar	Sandstone	Miocene	Alhama de Murcia, Crevillente	Regente (93267)
5	89368	**GORD**	Serra Grossa	Elche	359.3553	38.3106	284.3	forced-centering concrete pillar	Sandstone	Miocene	Crevillente	UJ/UA: 7003 Regente (89368)
6	93442	**LOMA**	La Loma	Murcia	358.9699	37.8705	244.6	forced-centering concrete pillar	Limestone	Quaternary	Bajo Segura	UJ/UA: 7008 Regente (93442)
7	95364	**MANI**	Manilla	Totana (Murcia)	358.3769	37.7424	985.1	forced-centering concrete pillar	Limestone	Miocene	Alhama de Murcia	Regente (95364)
8	87134	**SERR**	Serreta Larga	Novelda	359.2660	38.4050	408.8	forced-centering concrete pillar	Limestone	Jurassic	Alhama de Murcia	UJ/UA: 7001 Regente (87134)

**Table 6:** List of 56 Rinex files included in dataset S2 named: “UB_UJ_UA_REGENTE-2009-2024.tar.gz” available online. LOMA (i.e. 7003) data given in red were collected by the University of Jaén (UJ) and University of Alicante (UA) and for this reason are also included in dataset S3.

**2009** abad2600.09d.gz abad2610.09d.gz abad2620.09d.gz gord2590.09d.gz gord2600.09d.gz gord2610.09oserr2610.09d.gz aina2630.09d.gz serr2630.09d.gz aina2640.09d.gz crca2640.09d.gz aina2650.09d.gz crca2650.09d.gz aina2660.09d.gz crca2660.09o **2011** mani1430.11d.gz mani1440.11d.gz **2013** cabe0740.13d.gz cabe0750.13d.gz **2020** loma0200.20d.gz loma0210.20d.gz loma0220.20d.gz loma0230.20d.gz **2021** loma0250.21d.gz loma0260.21d.gz loma0270.21d.gz loma0280.21d.gz **2022** loma0110.22d.gz loma0120.22d.gz loma0130.22d.gz loma0140.22o **2023** loma0160.23d.gz loma0170.23d.gz loma0180.23d.gz loma0190.23d.gz **2024** abad0870.24d.gz abad0880.24d.gz gord0530.24d.gz aina0540.24d.gz gord0540.24d.gz aina0550.24d.gz gord0550.24d.gz mani0660.24d.gz mani0670.24d.gz crca0870.24d.gz crca0880.24d.gz crca0890.24d.gz loma0900.24d.gz loma0910.24d.gz cabe0920.24d.gz loma0920.24d.gz cabe0930.24d.gz serr0930.24d.gz cabe0940.24d.gz serr0940.24d.gz serr0950.24d.gz

These 8 REGENTE sites were observed by the University of Barcelona (UB) team during the four campaigns conducted in 2009, 2011, 2013 and 2024, which resulted in 40 daily observation files, with an observation duration of 5 to 24 h per day. Point LOMA (also referred to as 7008 by UJ and UA) was also observed by the University of Jaén group, in collaboration with the University of Alicante, during the campaigns of 2020, 2021, 2022 and 2023 (shown in red in Fig. 2). These 16 files were also included in the provided dataset, since they have not been published previously. On the other hand, data from the years 1999, 2001, 2002 and 2013 collected by the UJ/UA teams, which were also used in the paper, have already been published [8] and are available online to the public [11]. For this reason, they are not included here.

The provided 56 RINEX (40 UB; 16 UJ/UA) observation files are listed in Table 8.

As with dataset S1, we performed a quality control (QC) of these data using TEQC software [6] and provide the results in a file “***UB_UJ_UA_Regente_QCstats-2009-2024.txt***”. The output format of this file are described in Table 4.

### 3.3 Dataset S3: UJ-UA data from GESE-CV network (2019-2023).

This dataset includes 56 daily RINEX files from the GESE-CV (*Red Geodésica del SE de la Comunidad Valenciana*) network, which were acquired after the publication of the 2019 paper [8], covering 2019, 2020, 2021, 2022 and 2023 campaigns conducted by the University of Jaén and University of Alicante. Detailed description of these sites, together with the rest of the sites of the GESE-CV network, is provided in Table 7. Note that the site 7004 no longer exists and that 3 sites, 7001, 7003 and 7008, belong to the IGN REGENTE network and are respectively, referred to by the UB as: SERR, GORD and LOMA. Earlier data from these 3 REGENTE network sites, collected by the UB, are provided in dataset 2 described above.

**Table 7:** Location and the description of the GESE-CV geodetic network monuments of the UJ/UA. Data from the 3 stations of GESE-CV used in [1] and included in the given paper are shown in bold.

#	Num.	Code	Location	Municipality	Longitude (°)	Latitude (°)	MSLH (m)	Mnmnt. type	Rock Type	Rock Age	Related Faults	Notes
1	7001	SERR	Serreta Larga	Novelda	-0.734	38.405	408.8	forced-centering concrete pillar	Limestone	Cretaceous	Crevillente	UB: SERR Regente (87134)
2	7002	7002	El Tolomó	Hondón de las Nieves	-0.810	38.300	441.0	Concrete rect. Base w/ thread	Limestone	Cretaceous	Crevillente	
3	7003	GORD	Sierra Gorda	Elche	-1.014	38.311	284.3	Concrete rect. Base w/ thread	Sandstone	Miocene	Crevillente	UB: GORD Regente (89368)
4	7005	7005	Los Colorados	Abanilla	-1.014	38.180	228.9	Concrete rect. Base w/ thread	Limestone	Miocene	Crevillente	
5	7006	7006	San Isidro	Albatera	-0.847	38.165	86.0	Concrete rect. Base w/ thread	Dolomite	Triassic	Bajo Segura	
6	7007	7007	Santa Pola	Santa Pola	-0.517	38.209	197.8	Concrete rect. Base w/ thread	Limestone	Miocene	Bajo Segura	
**7**	**7008**	**LOMA**	**La Loma**	**Murcia**	**-1.030**	**37.871**	**244.6**	**Concrete rect. Base w/ thread**	**Limestone**	**Quaternary**	**Bajo Segura**	**UB: LOMA Regente (93442)**
8	7009	7009	Benejúzar	Benejúzar	-0.825	38.067	132.0	Concrete rect. Base w/ thread	Sandstone	Pliocene	Bajo Segura	
9	7010	7010	El Moncayo	Guardamar del Segura	-0.659	38.075	157.6	Concrete rect. Base w/ thread	Sandstone	Pliocene	Bajo Segura	
**10**	**7011**	**7011**	**La Vieja**	**San Miguel de Salinas**	**-0.791**	**37.949**	**229.8**	**Concrete rect. Base w/ thread**	**Sandstone**	**Miocene**	**Bajo Segura**	
**11**	**7112**	**7112**	**Portmán**	**Portmán**	**-0.833**	**37.608**	**228.7**	**Concrete rect. Base w/ thread**	**Dolomite**	**Triassic**	**Cartagena**	

The provided dataset S3 from the 3 stations listed in Table 8 in bold, include 54 RINEX observation files, which are available online [2], together with the datasets S1 and S2 described earlier. This daily RINEX files include new data from the 3 points of the GESE-CV network: 7008 (i.e., LOMA), 7011, 7112 collected by the UJ-UA, which were not used in the 2019 paper [8]. The rest of the data from the GESE-CV network, including data 1999, 2001, 2002 and 2013, are available online [11].

**Table 8:** List of 56 daily Rinex files included in the dataset S3 collected by the observed by the University of Jaén group, in collaboration with the University of Alicante during the 2019, 2020, 2021, 2022 and 2023 campaigns. The file containing data available online is called: “UJ_UA_GESE-CV_2019-2023.tar.gz” [2].

**2019**
71123290.19d.gz 71123300.19d.gz 71123310.19d.gz 71123320.19d.gz
**2020**
70110200.20d.gz loma0200.20d.gz 70110210.20d.gz loma0210.20d.gz 70110220.20d.gz loma0220.20d.gz 70110230.20d.gz loma0230.20d.gz 71123280.20d.gz 71123290.20d.gz 71123300.20d.gz 71123310.20d.gz
**2021**
70110250.21d.gz loma0250.21d.gz 70110260.21d.gz loma0260.21d.gz 70110270.21d.gz loma0270.21d.gz 70110280.21d.gz loma0280.21d.gz 71123250.21d.gz 71123260.21d.gz 71123270.21d.gz 71123280.21d.gz 71123290.21d.gz
**2022**
70110110.22d.gz loma0110.22d.gz 70110120.22d.gz loma0120.22d.gz 70110130.22d.gz loma0130.22d.gz 70110140.22d.gz loma0140.22d.gz 71123170.22d.gz 71123180.22d.gz 71123190.22d.gz 71123200.22d.gz 71123210.22d.gz
**2023**
70110160.23d.gz loma0160.23d.gz 70110170.23d.gz loma0170.23d.gz 70110180.23d.gz loma0180.23d.gz 70110190.23d.gz loma0190.23d.gz 71123180.23d.gz 71123190.23d.gz 71123200.23d.gz 71123210.23d.gz

As with the previous datasets, we performed a quality control (QC) for these data using TEQC software [6] and provide the results in a file “***UJ_UA_GESE-CV_QCstats-2009-2023.txt***”. The details of this file are described in Table 4.

## 4 Experimental Design, Materials and Methods

**Fig. 3:**
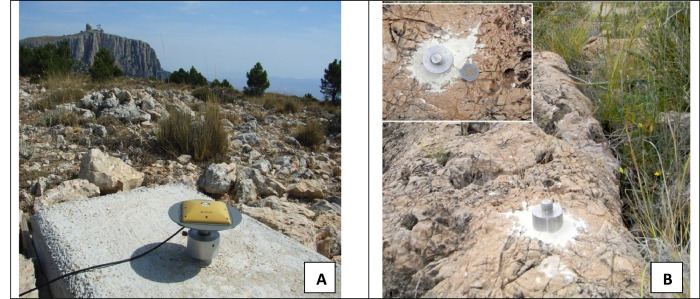
Photographs showing two examples of monuments of the CuaTeNeo network. Photos correspond to ESPU site (left) in Sierra Espuña (Murcia) and MOJA site (right) in Mojácar (Almería).

**Fig. 4:**
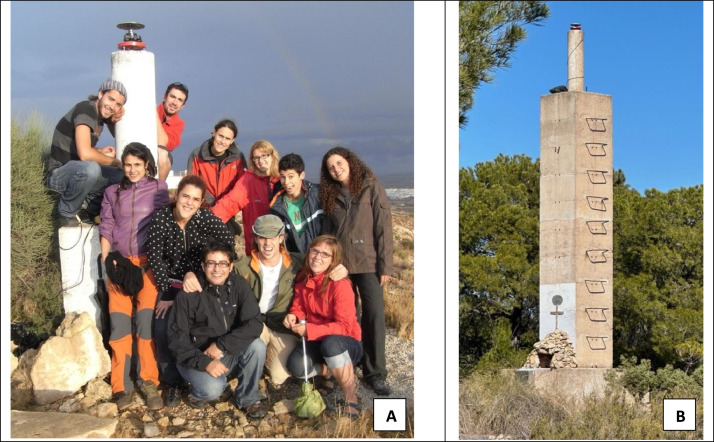
Example of the IGN REGENTE network monuments. A) Site GORD (Sierra Gorda between Alicante and Elche, https://datos-geodesia.ign.es/Red_Geodesica/Hoja0893/089368.pdf) during the 1999 campaign surrounded by the UB geology students, who volunteered for the for the field-work. B) Site MANI (Manilla, located in Sierra de Tercia, north of the city of Lorca, https://datos-geodesia.ign.es/Red_Geodesica/Hoja0953/095364.pdf). Photos of G. Khazaradze from 16/9/2009 and 6/3/2024, respectively.

As mentioned earlier, the provided 3 datasets include GNSS campaign observations from 3 different geodetic networks, covering the Eastern Betic Cordillera of Spain and covering the provinces of Alicante, Murcia and Almeria. The CuaTeNeo network was setup in 1996 with the specific aim of evaluating the earthquake-related hazard in this seismically active area of the Iberian Peninsula. The network consists of 15 highly stable monuments, covering ∼6000 km^2^ area in the Internal Zones of the Betic Cordillera, which consists of mountain ranges of metamorphic Palaeozoic and Mesozoic rocks separated by Neogene intermontane basins. 11 sites are constructed using a concrete monument (Fig. 3A) and 4 sites include only a special stainless mount directly epoxied inside the bedrock (Fig. 3B). Here we present the data of 8 stations (Table 2), since only these sites have been included in the accompanying study by Khazaradze et al. [1].

**Fig. 5:**
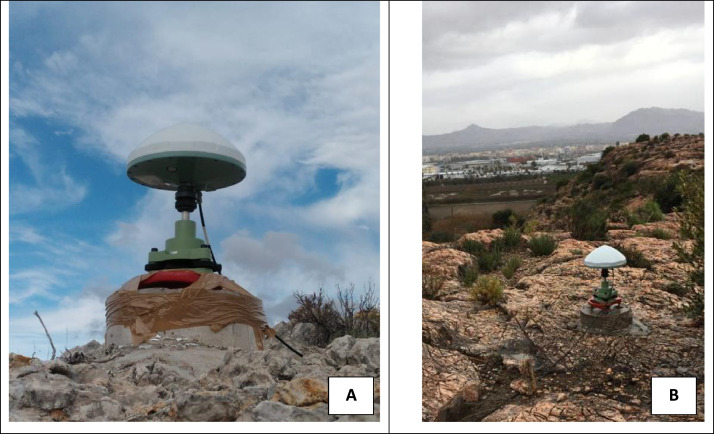
Example of GESE-CV geodetic network monuments . A) Site 7005 in Abanilla, Murcia. B) Site 7009 in Benejúzar, Alicante.

The REGENTE [10,12] geodetic network of the Spanish Geographic Institute IGN (www.ign.es) covers the whole country and was established in mid 1990s to provide a 3D geodetic reference frame with a high degree of accuracy. Overall, it includes more than 1000 monuments, but only 8 of them fall within the study area (Table 5). The REGENTE monuments are typically constructed using a cylindrical concrete pillar of 0.3 m in diameter and 1.2 m in height, with a forced centring system on the top, to ensure precise co-location of the GNSS antenna. In most cases, the concrete pilar is constructed over a rectangular concrete base, which can have a variable height and width. For example, the monument MANI (Manilla in Sierra Tercia, north of the town of Lorca) is more than 6 m high (Figs. 1 and Fig. 4, Fig. 5B). In contrast, the monument GORD (or 7003 in GESE-CV network) at Sierra Gorda (Alicante) has a total height of 2.2 m (Fig. 4A).

Finally, the dataset S3 includes data from GESE-CV (*Red Geodésica del SE de la Comunidad Valenciana*) network in Bajo Segura basin established by University of Jaén and University of Alicante in 1999 [11]. Detailed description of the 11 sites of the network is given in Table 7. This network includes 3 sites of the IGN REGENTE network: 7001, 7003 and 7008, which are respectively referred by the UB as SERR, GORD and LOMA. Earlier data from these 3 REGENTE network sites, collected by the UB, are provided in dataset 2 described above. The GESE-CV network includes 15 highly stable monuments, covering ∼10000 km^2^ in the Betic Cordillera.

## Limitations

No obvious limitations exist for the provided data. But as it is quite common for the GNSS campaign observations, some observations and consequently the provided RINEX files, have reduced duration time. Usually, all the daily observations have durations greater than 8 h, but in some cases, this time might be smaller. Most often, this is due to the draining of the external battery, used during the campaigns.

Additional limitation is the fact that the field log-sheets are not provided. Since the data cover almost 30 years of observations, in many cases the original field log-sheets are not anymore available. An extra effort was made to ensure that the information provided in the RINEX headers were correct. Especially, regarding the antenna types and heights, that are crucial during the post-processing.

## Ethics Statement

The authors confirm that they have read and followed the ethical requirements for publication in Data in Brief and confirm that the current work does not involve human subjects, animal experiments, or any data collected from social media platforms.

## CRediT authorship contribution statement

**Giorgi Khazaradze:** Conceptualization, Data curation, Writing – review & editing. **Pedro Alfaro:** Funding acquisition, Data curation. **Francisco Juan García-Tortosa:** Data curation. **Antonio Gil-Cruz:** Funding acquisition, Data curation. **Noemi Jacobo-Quiñones:** Data curation, Writing – review & editing. **Iván Martin-Rojas:** Funding acquisition, Data curation, Writing – review & editing. **Iván Medina-Cascales:** Data curation. **David Rodríguez Collantes:** Data curation.

## Data Availability

(Zenodo)GNSS data from the Eastern Betic Tectonic Arc (Spain) from multi-year campaigns (1997-2024) (Original data).

## References

[1] Khazaradze G., Jacobo-Quiñones N., Alfaro P., Medina-Cascales I., García-Tortosa F.J., Gil-Criz A.J., Rodríguez Collantes D., Martin-Rojas I. (2026). Tectonic salient characterization based on GNSS data: the case of the Eastern Betic Tectonic Arc in the western Mediterranean. Geod. Geodyn..

[2] G. Khazaradze, N. Jacobo-Quiñones, A.J. Gil, A. Staller, I. Medina-Cascales, I. Martin-Rojas, GNSS data from the Eastern Betic Tectonic Arc (Spain) from multi-year campaigns (1997-2024), (2025), doi: 10.5281/zenodo.17666304.

[3] Soro M., Giménez J., Fleta J. (1997).

[4] Gurtner W., Estey L. (2007). http://hdl.handle.net/10013/epic.38153.d001.

[5] Hatanaka Y. (2008). A compression format and tools for GNSS observation data. Bull. Geogr. Surv. Inst..

[6] Estey L., Meertens C.M. (1999). TEQC: the multi-purpose toolkit for GPS/GLONASS data. GPS Solut..

[7] Martínez Solares J.M., Mezcua J. (2002).

[8] Borque M.J., Sánchez-Alzola A., Martin-Rojas I., Alfaro P., Molina S., Rosa-Cintas S., Rodríguez-Caderot G., de Lacy C., Avilés M., Herrera-Olmo A., García-Tortosa F.J., Estévez A., Gil A.J. (2019). How much Nubia-Eurasia convergence is accommodated by the NE end of the Eastern Betic Shear Zone (SE Spain)? Constraints from GPS velocities. Tectonics.

[9] García-Mayordomo J., Insua-Arévalo J.M., Martínez-Díaz J.J., Jiménez-Díaz A., Martín-Banda R., Martín-Alfageme S., Álvarez-Gómez J.A., Rodríguez-Peces M., Pérez-López R., Rodríguez-Pascua M.A., Masana E., Perea H., Martín-González F., Giner-Robles J., Nemser E.S., Cabral J. (2012). The Quaternary active faults database of Iberia (QAFI v.2.0). J. Iber. Geol..

[10] Regidor Gutiérrez J., Prieto Morin J.F., Sanz Mejía J.M., Quirós Donate R., Barbadillo Fernández A. (2001). El Proyecto REGENTE. Topogr. Cartogr..

[11] Borque Arancón M.J., Sánchez Alzola A., Martin-Rojas I., Alfaro García P., Avilés M., Herrera-Olmo A.G., Tortosa F.J., Estévez Rubio A., Gil Cruz A.J. (2018). https://hdl.handle.net/10045/77387.

[12] Barandillo-Fernández A., Quirós-Donate R. (1996). Proyecto REGENTE Una nueva red geodésica Nacional. Fis. Tierra.

